# Seizure history and cognitive dysfunction in people with multiple sclerosis

**DOI:** 10.1177/13524585251326841

**Published:** 2025-03-18

**Authors:** David E Freedman, Jiwon Oh, Cecilia Meza, Anthony Feinstein

**Affiliations:** Department of Psychiatry, Temerty Faculty of Medicine, University of Toronto, Toronto, ON, Canada; Department of Psychiatry, Sunnybrook Health Sciences Centre, Toronto, ON, Canada; Division of Neurology, Temerty Faculty of Medicine, University of Toronto, Toronto, ON, Canada; Department of Medicine, Division of Neurology, St. Michael’s Hospital, Toronto, ON, Canada; Department of Psychiatry, Sunnybrook Health Sciences Centre, Toronto, ON, Canada; Department of Psychiatry, Temerty Faculty of Medicine, University of Toronto, Toronto, ON, Canada; Department of Psychiatry, Sunnybrook Health Sciences Centre, Toronto, ON, Canada

**Keywords:** Multiple sclerosis, outcome measurement, seizure, cognition, neuropsychology

## Abstract

**Background:**

Seizures are associated with reduced cognition in the general population and worse outcomes in people with multiple sclerosis (pwMS). Yet, it remains unclear whether seizures are linked to cognitive dysfunction in pwMS.

**Objectives:**

To evaluate the connection between seizure history and poorer cognition in pwMS.

**Methods:**

A consecutive sample of 803 pwMS reported any prior seizures. Covariates included age, sex, Wechsler Test of Adult Reading scores, educational years, Expanded Disability Status Scale (EDSS) scores, disease duration, disease subtype, high-efficacy disease-modifying therapy use, Hospital Anxiety and Depression Scale scores for anxiety and depression and Modified Fatigue Impact Scale scores. Linear regression analyses, controlling for covariates, were undertaken to predict Minimal Assessment of Cognitive Function in MS scores from seizure history.

**Results:**

Mean age was 44.01 years (*SD* = 11.58), 76.84% were female, and median EDSS was 2.0 (interquartile range (IQR) = 1.5–3.5). Accounting for covariates, people with seizures (*n* = 43, 5.35%) performed worse than those without (*n* = 760) on Judgement of Line Orientation (β = −0.09, *p* < 0.01), California Verbal Learning Test-II learning (β = −0.08, *p* < 0.01) and memory (β = −0.10, *p* < 0.01), Brief Visuospatial Memory Test-Revised learning (β = −0.08, *p* = 0.01) and memory (β = −0.07, *p* = 0.05), Symbol Digit Modalities Test (β = −0.06, *p* = 0.04), Paced Auditory Serial Addition Test (β = −0.10, *p* < 0.01) and Delis-Kaplan Executive Function System (β = −0.07, *p* = 0.02).

**Conclusions:**

A seizure history independently predicts reduced cognition in pwMS.

## Introduction

Cognitive dysfunction affects 40%–90% of people with multiple sclerosis (pwMS),^1,2^ and contributes to reduced quality of life and increased unemployment.^3,4^ It is thus important to identify those at risk of cognitive dysfunction in pwMS. Studies have suggested that reduced cognition in MS is associated with older age, male sex, low premorbid intelligence, decreased education, increased neurological disability, prolonged disease duration, disease course and depression, especially with comorbid anxiety.^1,5–7^ Cognition is also associated with whole brain atrophy, reduced grey matter volume, thalamic atrophy and an elevated total white matter lesion volume burden.^2,8,9^ Yet, apart from comorbid depression and anxiety,^7^ there are few independent modifiable targets to enhance cognition in pwMS.

Seizures could be one such target. Seizures occur in 1%–3% of pwMS, approximately two to three times more commonly than in the general population.^10,11^ Potential risk factors for seizures in MS include prolonged disease duration, increased disability, progressive illness, use of sphingosine-1-phosphate receptor modulators, brain atrophy, cortical involvement, temporal lobe lesions and thalamic dysfunction.^12–15^ Seizures are associated with adverse outcomes for pwMS, including elevated disease progression, reduced walking distance, decreased employment and increased mortality.^16,17^ However, little is known about whether there is a link between a history of seizures and cognition in pwMS.

In the general population, a history of seizures is linked to cognitive dysfunction.^18,19^ Although this relationship may be partially due to the effects of seizure frequency and severity (or inter-ictal activity) on cognition, other contributors include cognitive reserve, developmental factors, depression, brain pathology and the influence of anti-epileptic drugs.^19^ The complex association between seizures and cognition may be further complicated by the impact of MS.

In pwMS, studies have found elevated subjective cognitive concerns and objectively measured dysfunction in people with seizures and MS compared to those without seizures.^14,16,20,21^ However, these studies are limited by small sample size,^14,20,21^ lack of objective neuropsychological testing,^14,16^ the absence of an MS-validated neuropsychological battery^14,16,21^ or failing to adjust for potential confounding variables (e.g. level of neurological disability, disease duration, etc.).^14,16,20,21^ No study has evaluated whether a seizure history is independently linked to objectively measured cognitive dysfunction in pwMS. We address this gap in the MS literature with a large, consecutively recruited clinical sample. This study’s objective was to evaluate for an independent association between a seizure history and reduced cognition in pwMS.

## Methods

Participants were a consecutive sample of 803 adults with MS (assessed with the McDonald criteria)^22,23^ who completed neuropsychological testing as part of routine clinical care at a tertiary neuropsychiatry clinic in Toronto, Canada between 2020 and 2024. We followed the Strengthening the Reporting of Observational Studies in Epidemiology (STROBE) reporting guidelines, with the associated checklist for this study included as Supplementary Table 1.^24^

### Demographic and disease-related data

Demographic and disease-related data were documented from chart review and included age, sex, years of education, Expanded Disability Status Scale (EDSS),^25^ disease subtype, disease duration and use of disease-modifying therapy (DMT).

### Seizure history and cognitive data

A seizure history is routinely elicited by self-report as part of obtaining a history before neuropsychological testing. Any previous reported seizure constituted a seizure history (dichotomized by its presence or absence).

Cognition was assessed using raw scores from the Minimal Assessment of Cognitive Function in MS (MACFIMS), a neuropsychological battery designed and validated for pwMS.^26,27^ The MACFIMS includes tests of verbal fluency (Controlled Oral Word Association Test; COWAT), visuospatial function (Judgement of Line Orientation; JOLO), verbal learning and memory (California Verbal Learning Test – second edition; CVLT_TL for learning and CVLT_DR for delayed recall), visual learning and memory (Brief Visuospatial Memory Test – Revised; BVMT_TL for learning and BVMT_DR for delayed recall), processing speed (Symbol Digit Modalities Test; SDMT), working memory (Paced Auditory Serial Addition Test; two-second (PASAT_2 sec) and three-second (PASAT_3 sec) versions) and executive function (Delis-Kaplan Executive Function System; D-KEFS_CS for correct sorts score and D-KEFS_DS for descriptive score).^26^ Premorbid intelligence quotient was estimated by Wechsler Test of Adult Reading (WTAR) scores.^28^

Symptoms of anxiety and depression were recorded by the Hospital Anxiety and Depression Scale,^29^ previously validated for use in pwMS.^30^ We mean-centred HADS-A and HADS-D sub-scales and multiplied these scores to compute an interaction score (HADS-A × HADS-D), in keeping with prior work.^7^ Fatigue was measured with the Modified Fatigue Impact Scale (MFIS),^31^ also validated for use in pwMS.^32^

### Statistical analysis

Demographic, disease-related and cognitive data of people with or without a history of seizures were compared using *t*-tests and chi-square tests, where appropriate. Cognitive data comparison effect sizes were reported with the Hedge’s *g* statistic.

A linear regression analyses was used to assess whether a seizure history independently predicted decreased MACFIMS raw scores. Covariates included age, sex, WTAR scores, years of education, EDSS scores, disease duration, disease subtype (relapsing-remitting versus progressive MS), use of a high-efficacy DMT (e.g. ocrelizumab, ofatumumab, natalizumab or alemtuzumab), centred HADS-D and HADS-A scores, a HADS-A × HADS-D interaction and MFIS total score. These covariates were selected based on prior literature that suggested that these variables may influence rates of cognitive dysfunction or seizures in people with MS.^1,5–7,12–14^ To preserve the original data and in light of a small amount of missing data, participants with missing data in each analysis were excluded via listwise deletion. The significance threshold was set at *p* < 0.05. As no directly comparable study was available, based on the available sample size (*n* = 803), a desired power of 0.80, *p* < 0.05 and 13 predictor variables, the study was powered to detect standardized beta coefficients of 0.15 or above, equivalent to small effect sizes based on Cohen’s guidelines.^33^ Power calculations were conducted using R studio (pwr package).^34^

### Standard protocol approvals, registrations and patient consents

This study involving human participants was performed in line with the principles of the Declaration of Helsinki. The study was approved by the Research Ethics Board at Sunnybrook Health Sciences Centre, affiliated to the University of Toronto (Date: 30 December 2021/No. 5263). Data were collected as part of a retrospective chart review and a waiver of consent was obtained as part of the research ethics board approval process.

### Data availability

The dataset is available from the corresponding author upon reasonable request.

## Results

### Demographic and disease-related data

Of 803 participants, mean age was 44.01 years (*SD* = 11.58), years of education was 15.99 years (*SD* = 2.93), disease duration was 10.16 years (*SD* = 9.07), HADS-A score was 9.91 (SD = 4.32) and HADS-D score (7.14 (SD = 4.07). Median EDSS was 2.00 (interquartile range (IQR) = 1.50–3.50). Most participants were female (76.83%), had relapsing-remitting MS (83.69%) and took a DMT (66.06%). There were no significant differences between people with and without a seizure history regarding age, sex distribution, WTAR scores, years of education, EDSS scores, disease duration, disease subtype, use of DMT or scores on the HADS-A, HADS-D or MFIS. Table 1 describes demographic and disease-related data according to seizure history.

**Table 1.** Demographic and disease-related data stratified by history of seizures.

	No history of seizures (*n* = 760)		History of seizures (*n* = 43)		*t*
	*M*	*SD*	*M*	*SD*	
Age (years)	43.94	11.46	45.28	13.49	0.74
Years of education	15.98	2.90	16.16	3.44	0.40
Wechsler Test of Adult Reading (WTAR)	37.76	7.56	36.92	9.07	0.66
Expanded disability status scale (EDSS)	2.65	1.71	3.00	2.01	1.28
EDSS (Median and Interquartile Range)	2.0	1.5–3.5	2.5	2.0–3.5	
Disease duration (years)	10.07	8.99	10.84	10.48	0.54
HADS-A	9.89	4.31	10.31	4.54	0.61
HADS-D	7.09	4.02	7.98	4.94	1.37
Modified Fatigue Impact Scale (MFIS)	49.85	16.48	53.63	17.33	1.41
	*n*	%	*n*	%	χ^2^
Female	581/760	76.45	36/43	83.72	1.21
Disease subtype (RRMS vs PMS)					0.71
RRMS	638/760	83.95	34/43	79.07	
SPMS	59/760	7.76	5/43	11.63	
PPMS	63/760	8.29	4/43	9.30	
Any disease-modifying therapy	580/759	76.42	30/43	69.77	0.99
High-efficacy therapy (e.g. ocrelizumab, ofatumumab, natalizumab, alemtuzumab)	272/756	35.98	12/43	27.91	1.16

HADS-A: Hospital Anxiety and Depression Scale sub-scales for anxiety; HADS-D: Hospital Anxiety and Depression Scale sub-scales for depression; RRMS: relapsing-remitting MS; PMS: progressive MS; SPMS: secondary PMS; PPMS: primary PMS.

* *p* < 0.05; **p < 0.01.

**Figure 1.**
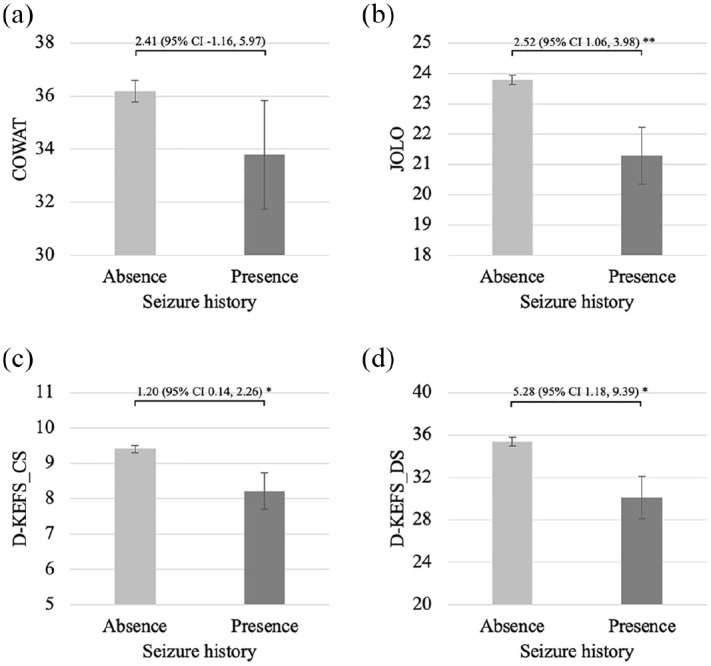
Comparisons between people with and without a seizure history on raw scores of tests of executive function and visuospatial processing. (a) COWAT, (b) JOLO, (c) D-KEFS_CS and (d) D-KEFS_DS. COWAT: Controlled Oral Word Association Test; JOLO: Judgement of Line Orientation; D-KEFS_CS: Delis-Kaplan Executive Function System correct sorts score; D-KEFS_DS: Delis-Kaplan Executive Function System descriptive score. Error bars indicate sub-group standard errors. Bracket and associated numbers reflect the sample mean difference and associated 95% confidence interval (95% CI). *p < 0.05; **p < 0.01.

**Figure 2.**
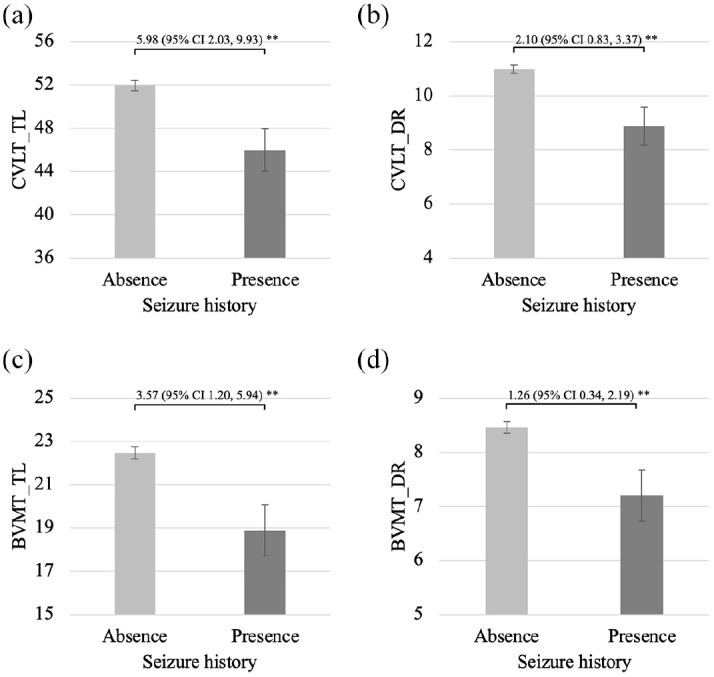
Comparisons between people with and without a seizure history on raw scores of tests of learning and memory. (a) CVLT_TL, (b) CVLT_DR, (c) BVMT_TL and (d) BVMT_DR. CVLT_TL: California Verbal Learning Test (second edition) total learning; CVLT_DR: California Verbal Learning Test (second edition) delayed recall; BVMT_TL: Brief Visuospatial Memory Test-revised total learning; BVMT_DR: Brief Visuospatial Memory Test-revised delayed recall. Error bars indicate sub-group standard errors. Bracket and associated numbers reflect the sample mean difference and associated 95% confidence interval (95% CI). *p < 0.05; **p < 0.01.

**Figure 3.**
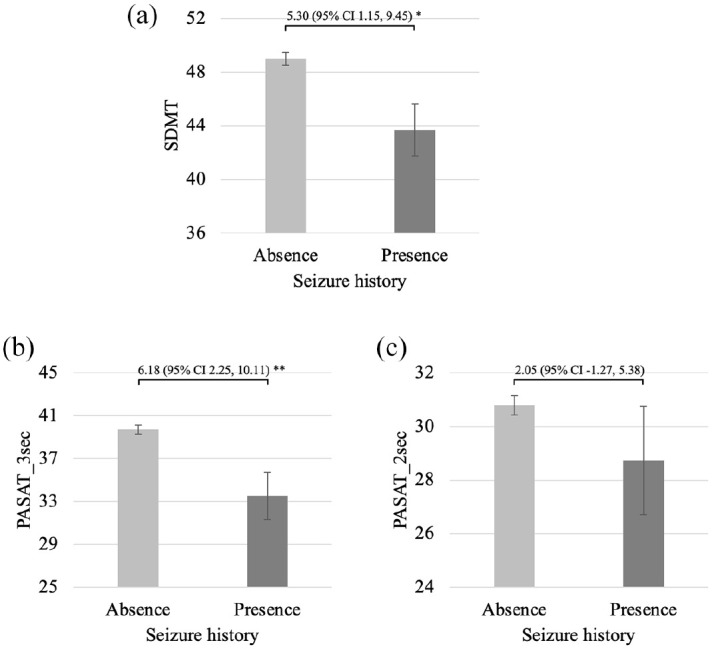
Comparisons between people with and without a seizure history on raw scores of tests of processing speed and working memory. (a) SDMT, (b) PASAT_3 sec and (c). PASAT_2 sec. SDMT: Symbol Digit Modalities Test; PASAT_3 sec: Paced Auditory Serial Addition Test 3 seconds; PASAT_2 sec: Paced Auditory Serial Addition Test 2 seconds. Error bars indicate sub-group standard errors. Bracket and associated numbers reflect the sample mean difference and associated 95% confidence interval (95% CI). *p < 0.05; **p < 0.01.

As described in Table 2, people with a seizure history performed more poorly than those without a history of seizures on the JOLO (Hedge’s *g* = 0.55, *p* < 0.01), CVLT_TL (Hedge’s *g* = 0.47, *p* < 0.01), CVLT_DR (Hedge’s *g* = 0.49, *p* < 0.01), BVMT_TL (Hedge’s *g* = 0.47, *p* < 0.01), BVMT_DR (Hedge’s *g* = 0.43, *p* < 0.01), SDMT (Hedge’s *g* = 0.41, *p* = 0.01), PASAT_3 sec (Hedge’s *g* = 0.51, *p* < 0.01), D-KEFS_CS (Hedge’s *g* = 0.44, *p* = 0.03) and D-KEFS_DS (Hedge’s *g* = 0.48, *p* = 0.01). There were no significant between-group differences for COWAT or PASAT_2 sec raw scores between participants with previous seizures or not. Figures 1–3 illustrate comparisons in cognitive raw scores between people with or without prior seizures.

**Table 2.** Comparisons in minimal assessment of cognitive function in multiple sclerosis raw scores between people with and without a history of seizures.

	No history of seizures			History of seizures			*t*	Hedges’ *g*
	*n*	*M*	*SD*	*n*	*M*	*SD*		
COWAT	756	36.19	11.36	42	33.79	13.31	−1.32	0.21
JOLO	748	23.79	4.50	40	21.28	5.94	−3.38**	0.55
CVLT_TL	759	51.96	12.82	43	45.98	12.94	−2.97**	0.47
CVLT_DR	757	10.98	4.05	42	8.88	4.56	−3.25**	0.49
BVMT_TL	749	22.47	7.54	41	18.90	7.48	−2.95**	0.47
BVMT_DR	749	8.46	2.94	41	7.20	3.03	−2.67**	0.43
SDMT	749	48.98	13.07	40	43.68	12.35	−2.51*	0.41
PASAT_3 sec	723	39.70	12.09	39	33.51	13.79	−3.09**	0.51
PASAT_2 sec	658	30.78	9.38	33	28.73	11.63	−1.21	0.22
D-KEFS_CS	753	9.41	2.71	42	8.21	3.35	−2.28*,^a^	0.44
D-KEFS_DS	753	35.40	10.84	42	30.12	12.95	−2.59*,^a^	0.48

*n*: Number of participants; COWAT: Controlled Oral Word Association Test; JOLO: Judgement of Line Orientation; CVLT_TL: California Verbal Learning Test (second edition) total learning; CVLT_DR: California Verbal Learning Test (second edition) delayed recall; BVMT_TL: Brief Visuospatial Memory Test-Revised total learning; BVMT_DR: Brief Visuospatial Memory Test-Revised delayed recall; SDMT: Symbol Digit Modalities Test; PASAT_3 sec: Paced Auditory Serial Addition Test 3 seconds; PASAT_2 sec: Paced Auditory Serial Addition Test 2 seconds; D-KEFS_CS: Delis-Kaplan Executive Function System correct sorts score; D-KEFS_DS: Delis-Kaplan Executive Function System descriptive score.

a Significant Levene’s test, and thus, Welch’s *t*-test used. Student’s *t*-test used in all other comparisons.

* *p* < 0.05; **p < 0.01.

After adjusting for covariates, seizure history was an independent predictor of decreased scores on the JOLO (β = −0.09, *p* < 0.01), CVLT_TL (β = −0.08, *p* < 0.01), CVLT_DR (β = −0.10, *p* < 0.01), BVMT_TL (β = −0.08, *p* = 0.01), BVMT_DR (β = −0.07, *p* = 0.05), SDMT (β = −0.06, *p* = 0.04), PASAT_3 sec (β = −0.10, *p* < 0.01) and D-KEFS_DS (β = −0.07, *p* = 0.02). Seizure history did not independently predict COWAT, PASAT_2 sec and D-KEFS_CS raw scores. Table 3 includes all of the standardized beta coefficients from these regression models.

**Table 3.** Standardized beta coefficients from linear regression analyses to predict minimal assessment of cognitive function in multiple sclerosis raw scores from seizure history, adjusted for covariates.

	Standardized beta coefficients													*R* ^2^
	Sz. Hx.	Age	Sex	WTAR	Edu.	EDSS	Dx. Dur.	RMSv PMS	HET use	HADS-D	HADS-A	Inter.	MFIS	
COWAT	−0.01	0.07	−0.01	0.42**	0.04	−0.18**	0.00	0.02	0.03	−0.10*	0.05	−0.05	−0.07	0.27
95% CI LL	−0.08	−0.01	−0.07	0.35	−0.03	−0.27	−0.09	−0.06	−0.04	−0.18	−0.02	−0.11	−0.15	
95% CI UL	0.05	0.14	0.06	0.49	0.10	−0.10	0.10	0.10	0.09	−0.01	0.13	0.02	0.02	
JOLO	−0.09**	−0.10*	−0.20**	0.35**	−0.04	−0.15**	−0.02	0.05	0.00	0.03	−0.05	−0.07*	−0.09*	0.21
95% CI LL	−0.15	−0.18	−0.27	0.29	−0.11	−0.24	−0.10	−0.03	−0.08	−0.06	−0.13	−0.14	−0.17	
95% CI UL	−0.02	−0.02	−0.13	0.42	0.03	−0.07	0.05	0.14	0.09	0.12	0.03	−0.01	0.00	
CVLT_TL	−0.08**	−0.11**	0.19**	0.21**	0.18**	−0.26**	−0.06	0.03	0.01	−0.06	0.04	−0.05	−0.05	0.31
95% CI LL	−0.14	−0.18	0.12	0.15	0.12	−0.34	−0.14	−0.05	−0.06	−0.14	−0.04	−0.11	−0.13	
95% CI UL	−0.02	−0.03	0.25	0.28	0.25	−0.18	0.01	0.10	0.07	0.03	0.11	0.01	0.04	
CVLT_DR	−0.10**	−0.09*	0.16**	0.20**	0.16**	−0.23**	−0.10*	0.02	−0.03	−0.10*	0.06	−0.05	−0.03	0.27
95% CI LL	−0.16	−0.16	0.09	0.14	0.09	−0.31	−0.18	−0.06	−0.10	−0.19	−0.01	−0.11	−0.08	
95% CI UL	−0.03	−0.01	0.22	0.27	0.22	−0.14	−0.03	0.10	0.04	−0.01	0.14	0.02	0.04	
BVMT_TL	−0.08*	−0.26**	0.02	0.28**	0.02	−0.19**	−0.07	0.03	0.00	0.03	0.00	−0.06	−0.07	0.26
95% CI LL	−0.14	−0.33	−0.04	0.21	−0.05	−0.28	−0.15	−0.01	−0.08	−0.06	−0.07	−0.12	−0.15	
95% CI UL	−0.02	−0.18	0.09	0.35	0.09	−0.11	0.01	0.11	0.07	0.12	0.07	0.00	0.02	
BVMT_DR	−0.07*	−0.23**	0.03	0.26**	0.02	−0.19**	−0.07	0.06	0.01	0.02	0.04	−0.05	−0.08	0.23
95% CI LL	−0.13	−0.31	−0.04	0.19	−0.05	−0.27	−0.15	−0.02	−0.06	−0.07	−0.04	−0.11	−0.17	
95% CI UL	0.00	−0.14	0.09	0.33	0.09	−0.10	0.01	0.14	0.08	0.10	0.12	0.02	0.01	
SDMT	−0.06*	−0.18**	0.05	0.22**	0.02	−0.32**	−0.07	0.03	−0.03	−0.01	0.12*	−0.09**	−0.13**	0.33
95% CI LL	−0.12	−0.26	−0.01	0.16	−0.05	−0.40	−0.14	−0.04	−0.09	−0.09	0.04	−0.15	−0.21	
95% CI UL	0.00	−0.11	0.11	0.29	0.08	−0.24	0.00	0.11	0.04	0.08	0.19	−0.03	−0.05	
PASAT_3 sec	−0.10**	−0.03	−0.13**	0.31**	0.08*	−0.22**	0.01	0.09*	0.01	0.02	0.00	−0.03	−0.09	0.20
95% CI LL	−0.16	−0.11	−0.20	0.24	0.00	−0.31	−0.07	0.00	−0.07	−0.07	−0.07	−0.10	−0.18	
95% CI UL	−0.03	0.06	−0.06	0.39	0.15	−0.13	0.09	0.17	0.08	0.11	0.07	0.04	0.00	
PASAT_2 sec	−0.03	0.02	−0.13**	0.23**	0.11**	−0.15**	−0.04	0.03	0.00	−0.01	0.03	−0.03	−0.14**	0.15
95% CI LL	−0.10	−0.07	−0.20	0.15	0.03	−0.25	−0.13	−0.06	−0.08	−0.11	−0.06	−0.10	−0.24	
95% CI UL	0.05	0.11	−0.05	0.31	0.19	−0.05	0.04	0.01	0.08	0.09	0.12	0.05	−0.04	
D-KEFS_CS	−0.06	−0.16**	−0.08*	0.29**	0.17**	−0.24**	0.01	0.04	0.04	0.07	−0.07	−0.02	−0.02	0.26
95% CI LL	−0.13	−0.24	−0.14	0.22	0.10	−0.33	−0.07	−0.05	−0.03	−0.01	−0.14	−0.08	−0.09	
95% CI UL	0.00	−0.08	−0.01	0.36	0.24	−0.016	0.09	0.12	0.11	0.16	0.01	0.04	0.06	
D-KEFS_DS	−0.07*	−0.13**	−0.06	0.34**	0.14**	−0.24**	−0.01	0.04	0.02	0.07	−0.06	−0.02	−0.01	0.27
95% CI LL	−0.14	−0.21	−0.12	0.27	0.07	−0.33	−0.09	−0.04	−0.05	−0.02	−0.13	−0.09	−0.10	
95% CI UL	−0.01	−0.06	0.00	0.40	0.21	−0.16	0.07	0.12	0.09	0.15	0.02	0.04	0.07	

Sz. Hx.: seizure history; WTAR: Wechsler Test of Adult Reading; Edu.: years of education; EDSS: Expanded Disability Status Scale; Dx. Dur.: disease duration; RMSvPMS: Relapsing-Remitting Multiple Sclerosis versus Progressive Multiple Sclerosis; HET: high-efficacy therapy; HADS-D: Centred Hospital Anxiety and Depression Scale sub-scales for depression; HADS-A: Centred Hospital Anxiety and Depression Scale sub-scales for anxiety; Inter.: Interaction between depression and anxiety; MFIS: Modified Fatigue Impact Scale; COWAT: Controlled Oral Word Association Test; 95% CI: 95% confidence interval; LL: lower limit; UL: upper limit; JOLO: Judgement of Line Orientation; CVLT_TL: California Verbal Learning Test (second edition) total learning; CVLT_DR: California Verbal Learning Test (second edition) delayed recall; BVMT_TL: Brief Visuospatial Memory Test-Revised total learning; Brief Visuospatial Memory Test-Revised total learning delayed recall; SDMT: Symbol Digit Modalities Test; PASAT_3 sec: Paced Auditory Serial Addition Test 3 seconds; PASAT_2 sec: Paced Auditory Serial Addition Test 3 seconds; D-KEFS_CS: Delis-Kaplan Executive Function System correct sorts score; D-KEFS_DS: Delis-Kaplan Executive Function System descriptive score.

* *p* < 0.05; **p < 0.01.

## Discussion

Findings from a consecutive sample of 803 pwMS revealed that individuals with a self-reported history of seizures were more impaired than those without across multiple cognitive domains. These findings are in keeping with the non-MS literature,^18,19^ and while similar results have been reported in a smaller MS literature,^20,21^ our study is, to the best of our knowledge, the first to demonstrate that a history of seizures per se is an independent predictor of cognitive dysfunction in pwMS. We examine this finding in detail and discuss its potential implications for future research and clinical care.

Only two studies have examined the association between seizure history and objectively measured cognition in pwMS.^20,21^ In a 3-year case–control longitudinal study of 92 people with relapsing-remitting MS, Calabrese and colleagues found that those with epilepsy (case) demonstrated worse cognitive performance and faster cognitive decline compared to people without epilepsy (control).^20^ In another study (including only 10 participants who had seizures), Uribe-San-Martin and colleagues found a link between poor seizure control and reduced cognitive performance.^21^ However, both of these studies had small samples and did not control for the influence of potential confounding demographic, disease-related or neuropsychiatric variables.^20,21^

There are several potential explanations for this association. Ictal or inter-ictal activity could worsen cognition, aligning with the general population literature,^18^ and evidence from a small sample of pwMS showing that adequate seizure control improves cognitive performance.^21^ Alternatively, MS-related brain changes may increase the risk of both seizures and cognitive dysfunction. For example, cortical pathology is linked to seizure history and cognitive dysfunction in pwMS.^2,13,14,20,21^ In pwMS who have epilepsy, temporal lobe seizures are common^13,14^ and associated with severe hippocampal damage.^13^ In relapsing-remitting MS, epilepsy was associated with increased cortical lesions, decreased cortical thickness, and a faster decline in grey matter fraction over 3 years compared to people without epilepsy.^20^ Furthermore, in pwMS who have seizures, poor seizure control was linked to reduced brain volume and cortical lesions.^21^ Together, these early data suggest a connection between seizure history and cortical pathology in pwMS. Longitudinal studies will be needed to clarify the temporal order of the connection between seizures and cognition in pwMS.

As previously noted, seizures occur in approximately 1%–3% of pwMS.^10,11^ Despite our sample being skewed towards milder disability, there is a higher prevalence of seizures (5.35%) in our sample relative to the published literature. This, therefore, suggests another potential source of bias – pwMS are referred because of neurologists’ concerns about cognition. A sample biased towards cognitive impairment would by reverse association, be expected to have a higher percentage of people with seizures, as seen in our sample. This emphasizes the importance of routine questions about seizure history in MS neuropsychiatric evaluations, particularly in light of the potential for several psychotropic medications to alter the seizure threshold.

There are a few limitations of this study to consider. Given the low prevalence of seizure disorders in pwMS and that only a small proportion of the cohort had seizures, it is possible that there was a lack of power to detect differences. Nonetheless, this is one of the largest cohorts where this question has been evaluated and there were no significant differences in demographic, disease-related or neuropsychiatric data between those with or without seizures. As such, further study is required. Our sample is biased towards people with relapsing illness. Although this is typical of a tertiary neuropsychiatry clinic,^7^ seizures occur more commonly in people with progressive illness and future studies should bolster enrolment of these individuals.^12^ Notably, we did not find an association between seizure history and progressive disease in the preliminary Chi-square test possibly due to a small sample size. Furthermore, accounting for disease subtype did not affect the relationship between seizure history and cognition. We also lack neuroimaging, electroencephalographic or recent disease activity data, or data on socioeconomic variables or medical comorbidities. These data could help clarify whether neuro-anatomical or ictal abnormalities, ongoing disease activity or other variables account for the link between seizure history and cognition.

In addition, this study relied on self-report of seizure history. In this context, we lack data about seizure semiology, classification or timeline (e.g. age at seizure occurrence, number of seizures, duration of seizures). Yet, all participants in this study were previously assessed and referred by neurologists, potentially enhancing the accuracy of self-reported seizure histories. Nevertheless, it remains unknown whether seizures were active at the time of neuropsychological testing or whether participants were taking specific anti-epileptic medications such as phenytoin or phenobarbital, which some studies,^35^ but not all,^18^ suggest are linked to increased risk of cognitive side effects. Future studies could build upon this work by verifying seizure histories and gathering comprehensive seizure-related data to clarify whether these factors influence the relationship between seizure history and cognition.

In conclusion, in a large consecutive clinical sample, our data show that a seizure history is associated with cognitive dysfunction in pwMS, independent of demographic data, disease-related variables or neuropsychiatric symptoms. Clinicians could gather a history of seizures to identify those at elevated risk of impaired cognition for comprehensive neuropsychological assessments. While this study cannot disentangle the influences of seizure activity, neuro-anatomical factors and anti-epileptic medications in contributing to this link, it sets up the foundation for the studies that can. Identifying modifiable contributors remains important to reducing cognitive dysfunction in MS. Seizures may be one such factor. At this stage, it is unclear whether improving seizure control would enhance cognition in pwMS; however, our study opens a potential avenue for future exploration.

## Supplemental Material

sj-docx-1-msj-10.1177_13524585251326841 – Supplemental material for Seizure history and cognitive dysfunction in people with multiple sclerosis Supplemental material, sj-docx-1-msj-10.1177_13524585251326841 for Seizure history and cognitive dysfunction in people with multiple sclerosis by David E Freedman, Jiwon Oh, Cecilia Meza and Anthony Feinstein in Multiple Sclerosis Journal
