# Supplementary material for: Seizure history and cognitive dysfunction in people with multiple sclerosis
Source: Mult Scler. 2025 Mar 18;31(6):668–76. doi: 10.1177/13524585251326841 (PMC12092947; doi:10.1177/13524585251326841)
Supplement: sj-docx-1-msj-10.1177_13524585251326841 – Supplemental material for Seizure history and cognitive dysfunction in people with multiple sclerosis [file sj-docx-1-msj-10.1177_13524585251326841.docx]

**Supplementary Table 1.** STROBE checklist for cross-sectional studies adapted for this study.

|  | Item No | Page No |
| --- | --- | --- |
| **Title and abstract** | 1 | 1 |
|  |  | 1 |
| Introduction | | |
| Background/rationale | 2 | 1, 2 |
| Objectives | 3 | 2 |
| Methods | | |
| Study design | 4 | 2 |
| Setting | 5 | 2 |
| Participants | 6 | 2 |
| Variables | 7 | 2 |
| Data sources/ measurement | 8* | 2 |
| Bias | 9 | 2, 3 |
| Study size | 10 | 2, 3 |
| Quantitative variables | 11 | 2, 3 |
| Statistical methods | 12 | 2, 3 |
|  |  | N/A |
|  |  | 2 |
|  |  | N/A |
|  |  | N/A |
| Results | | |
| Participants | 13* | 3 |
|  |  | 4 |
|  |  | N/A |
| Descriptive data | 14* | 3 |
|  |  | 3, 4 |
| Outcome data | 15* | 4 |
| Main results | 16 | 3, 4 |
|  |  | N/A |
|  |  | N/A |
| Other analyses | 17 | N/A |
| Discussion | | |
| Key results | 18 | 4 |
| Limitations | 19 | 5, 7, 8 |
| Interpretation | 20 | 4, 5, 7, 8 |
| Generalisability | 21 | 8 |
| Other information | | |
| Funding | 22 | 8 |
